# Microbial Community Succession and Response to Environmental Variables During Cow Manure and Corn Straw Composting

**DOI:** 10.3389/fmicb.2019.00529

**Published:** 2019-03-18

**Authors:** Qingxin Meng, Wei Yang, Mengqi Men, Ayodeji Bello, Xiuhong Xu, Benshu Xu, Liting Deng, Xin Jiang, Siyuan Sheng, Xiaotong Wu, Yue Han, Haifeng Zhu

**Affiliations:** College of Resources and Environment, Northeast Agricultural University, Harbin, China

**Keywords:** cow manure, composting, high throughput sequencing, microbial community, environmental factor

## Abstract

In composting system, the composition of microbial communities is determined by the constant change in the physicochemical parameters. This study explored the dynamics of bacterial and fungal communities during cow manure and corn straw composting using high throughput sequencing technology. The relationships between physicochemical parameters and microbial community composition and abundance were also evaluated. The sequencing results revealed the major phyla included Proteobacteria, Bacteroidetes, Firmicutes, Chloroflexi and Actinobacteria, Ascomycota, and Basidiomycota. Linear discriminant analysis effect size (LEfSe) illustrated that Actinomycetales and Sordariomycetes were the indicators of bacteria and fungi in the maturation phase, respectively. Mantel test showed that NO_3_^-^-N, NH_4_^+^-N, TN, C/N, temperature and moisture content significantly influenced bacterial community composition while only TN and moisture content had a significant effect on fungal community structure. Structural equation model (SEM) indicated that TN, NH_4_^+^-N, NO_3_^-^-N and pH had a significant effect on fungal abundance while TN and temperature significantly affected bacterial abundance. Our finding increases the understanding of microbial community succession in cow manure and corn straw composting under natural conditions.

## Introduction

Huge amount of crop residues and livestock manure are produced in China every year. However, dealing with these increasing volumes of agricultural wastes is becoming a major problem, because most of the wastes generated are not treated or efficiently recycled (Huang et al., 2013; Renaud et al., 2017). Notably, untreated cow manure has caused serious environmental pollution, and this is inimical to human health and the general wellbeing of a community. Thus, there is a need for effective management of these agricultural wastes. Composting is considered to be an effective method for disposal of agricultural solid waste and the final product is suitable for agricultural and horticultural use as part of a sustainable strategy (Neher et al., 2013; Kang et al., 2014; López-González et al., 2015).

Composting is a self-heating, dynamic, and complex biochemical process, during which the successful bio-transformation of organic substrates is completed by many different microorganisms including bacteria and fungi (Hultman et al., 2010; Awasthi et al., 2017). The bacterial communities in the composting system have been reported in many studies, which exert important roles in the degradation of organic matter, proteins, lipids, cellulose, and lignin in the composting process (Karadag et al., 2013; Ren et al., 2016). In addition, fungi also play an important role in composting. They have the ability to use many carbon substrates as a food source and survive under dry, acidic, low level nitrogen conditions and a large number of fungal species have been found in the mesophilic stage of composting (Ryckeboer et al., 2003; Bonito et al., 2010). Indeed, the complex action of a large number of microorganisms is directly affected by various environmental factors of composting, such as temperature, moisture, carbon/nitrogen ratio, oxygen rate, and pH, among others (Insam et al., 2010). Temperature is one of the main factors controlling the composting reaction rate due to its effect on the microbial metabolic rate and population structure, which defines the different phases of composting (Neher et al., 2013; Huhe et al., 2017). Successions of microbial communities are closely related to the duration of the composting process and the quality of the compost (Tian et al., 2013).

Numerous studies have assessed the microbial community dynamics in the composting process using both culture-dependent and culture-independent methods (Chow et al., 2013; Petersen et al., 2015). However, we still have limited understanding of microbial community structures, especially fungal communities, in composting process, in relation to the microbial complexity and the limitation of detection methods (Wang et al., 2018b). Nowadays, the high-throughput sequencing technology, which has the ability to detect more microorganisms than other methods (Petrosino et al., 2009), is the most frequently used molecular method for evaluating microbial diversity and community structure (Mishra et al., 2014). Therefore, the dynamics of microbial communities in various composting systems have been studied, such as in cow manure and wood chips (Wang et al., 2018b), corn cobs and fresh cow dung (Zhang et al., 2016) also vegetable waste and cow dung (Varma et al., 2018). Thus, it is necessary to evaluate the microbial community succession in composting using high-throughput sequencing technology.

In this study, composting was constructed using cow manure and corn straw under natural conditions. This study is aimed to determine the variation of bacterial and fungal communities during the composting process using high-throughput sequencing technology, also to confirm the correlations between environmental factors and bacterial-fungal community structures and abundances.

## Materials and Methods

### Composting Process and Sampling

Three natural composting piles containing cow manure and corn straw were prepared at a ratio of 5:1 in Harbin, Northeast China in July, 2017. Composting piles approximately 2.5 m × 1.5 m × 1.5 m (length × width × height) with the corn straw acting as the bulking material. The composting piles had about 65% moisture content and 30:1 C/N ratio, the characteristics of the raw materials are shown in Table 1. Three artificial turnings were conducted on day 8, 23, and 40 within 59 days of composting. The sub-samples were collected from nine different points at three depths (20 cm, 70 cm, 120 cm from the top) of the composting piles on day 0, 2, 5, 8, 12, 18, 39, 49, and 59. The sub-samples were mixed and sub-divided into two samples. One was stored at -80°C for the bacterial and fungal DNA extraction and the other sample was sub-divided into two for the measurement of ammonium and nitrate stored at 4°C. The remaining sample was air-dried and prepared for the physicochemical analysis. The samples collected on day 0, 2, 18, 49, and 59 were then selected to represent initial, mesophilic, thermophilic, cooling, and maturation phases.

**Table 1:** The physicochemical characteristics of the raw materials.

Materials	TN (g/kg)	C/N ratio	NO_3_^-^-N (mg/kg)	NH_4_^+^-N (mg/kg)	pH	Moisture content (%)
Cow manure	17.7	22.36	95.9	1036.2	9.05	70.57
Corn stalk	6.35	55.69	–^a^	–^a^	–^a^	9.3

*–^*a*^, not determined; TN, total nitrogen; C/N, the ratio of TC to TN.*

### Physicochemical Parameters Analysis

Temperature levels at the surface, core, and bottom of the composting piles were measured with digital thermometers daily and the average temperature on each sampling day was used for analysis. The environment temperature was also monitored near the composting piles. pH was determined after shaking the fresh samples in water at a ratio of 1:10 (w/v) at 120 r/min for 60 min and the moisture content was determined by oven-drying to a constant weight at 105°C (Abid and Sayadi, 2006). Dry combustion method was used to determine the content of the total organic carbon (TC), total nitrogen (TN) content was measured using the Kjeldahl method (Kimberly and Roberts, 1905; Abad et al., 2002) while Ammonium (NH_4_^+^-N) and nitrate (NO_3_^-^-N) were extracted with 2 mol/L KCl and analyzed by Dual channel flow analyzer (AA3, Germany).

### DNA Extraction, PCR Amplification, and Sequencing

Genomic DNA was extracted from compost samples described in Liu et al. (2011) and the extracted DNA was purified using a DNA gel purification kit (Omega, United States) according to the manufacturer’s instructions. The quality of DNA was examined by electrophoresis in 1.0% agarose gel and the concentration was measured with a spectrophotometer (NanoDrop 2000, United States). The primers 515F and 909R (Tu et al., 2017), ITS4 (Bokulich and Mills, 2013), and gITS7 (Ihrmark et al., 2012) were used to amplify 16S rRNA gene and ITS gene, respectively. PCR amplification was carried out in a final 25.0 μL reaction solution including 12.5 μL Taq-HS PCR Forest Mix, 0.2 μL each primer, 1.0 μL template DNA, and 11.1 μL ddH_2_O. For details of the thermal cycling conditions see the Supplementary Material. Purified PCR products with concentration >10 ng/μL and OD 260/OD 280≈1.8 were subjected to Illumina MiSeq platform at Environmental Genome Platform of Chengdu Institute of Biology, Chinese Academy of Sciences. The abundance of 16S rRNA gene and ITS gene was determined in triplicate using ABI-7500 Real-Time PCR systems. The 20.0 μL reaction mixtures containing 16.5 μL ChamQ SYBR Color qPCR Master Mix, 0.8 μL each primer and 2.0 μL template DNA. The raw sequence data had been deposited to the NCBI Sequence Read Archive with accession Nos. SRP150164 and SRP150167 for 16S rRNA gene and ITS gene, respectively.

### Bioinformatics Analysis

The low quality sequences (length <250 bp, ambiguous bases, or with an average quality score <25) were removed using QIIME Pipeline Version 1.8.0 (Caporaso et al., 2010) and chimeras were discarded using the “chimera.uchime” command in Mothur (Schloss et al., 2009) before further analysis. The chimera free reads were clustered into different operational taxonomic units (OTUs) at 97% similarity threshold using USEARCH v8.0 (Edgar, 2013) after dereplication and discarding all singletons. Representative sequences from each OTU of 16S rRNA gene were assigned using the GREENGENES database (McDonald et al., 2011), while ITS gene sequences were assigned using the UNITE database (Abarenkov et al., 2010). The sequences number of each sample was normalized to the smallest sample size using the “normalized.shared” command in Mothur (Schloss et al., 2009).

### Statistical Analysis

One-way ANOVA was used to determine the effects of composting period on NO_3_^-^-N, NH_4_^+^-N, TN, C/N, pH, temperature, moisture content, bacterial Shannon diversity index, fungal Shannon diversity index, 16S rRNA gene abundance and ITS gene abundance. Shannon diversity index (H) was calculated using the “diversity” function in the Vegan package (Oksanen et al., 2013) in R (R Development Core Team, 2015). Structural equation model (SEM) was used to detect the direct and indirect effect of physicochemical parameters on abundance of bacteria and fungi using AMOS (Arbuckle, 2011). Model adequacy was determined by χ^2^ tests (*P* > 0.05), goodness-of-fit index (GFI > 0.9), Akaike Information Criteria (AIC), and root square mean errors of approximation (RSMEA < 0.05, Hooper et al., 2008). Furthermore, bacterial and fungal community structure was visualized by non-metric multidimensional scaling (NMDS) with the Bray–Curtis dissimilarity matrices using the Vegan package (Oksanen et al., 2013) to elucidate dissimilarities in bacterial and fungal community composition among different composting stages. Mantel test was performed to assess the correlations between bacterial and fungal community structure and environmental variables using the ecodist package (Goslee and Urban, 2007), partial Mantel test was also performed using vegan package (Oksanen et al., 2013). Linear discriminant analysis (LDA) effect size (LEfSe) was applied to search for statistically different biomarkers between different composting stages (Segata et al., 2011). Pearson correlation between environmental variables and the main genera (relative abundance >1%) was also analyzed.

## Results

### Physicochemical Changes During Composting

The variation of physicochemical parameters is shown in Supplementary Figures S1, S2. One-way ANOVA analysis showed that physicochemical parameters changed significantly during the whole composting process (Supplementary Table S1). The pH increased rapidly during the first 18 days and dropped to stable value at the end of composting (Supplementary Figure S1A). The temperature in the piles increased spontaneously across both the mesophilic and thermophilic phases and was maintained above 55°C for 30 days until it decreased during the cooling and maturation stages (Supplementary Figure S1B). As shown in Supplementary Figure S1C, the moisture content dropped gradually across the days of composting process. The change in NO_3_^-^-N in the composting piles with respect to time is presented in Supplementary Figure S2A. The concentration of NO_3_^-^-N increased slightly during the first 18 days and increased rapidly toward the end of the composting process. NH_4_^+^-N decreased during the mesophilic phase of the composting process (Supplementary Figure S2B). Subsequently, NH_4_^+^-N contents increased at the thermophilic phase and then decreased across both the cooling and maturation phase. TN increased slowly during the initial stage but rapidly increased during the thermophilic phase and became stable toward the end of composting (Supplementary Figure S2C). The change of C/N was shown in Supplementary Figure S2D and it had opposite trend compared with TN.

### Microbial Diversity and Abundance

**FIGURE 1:**
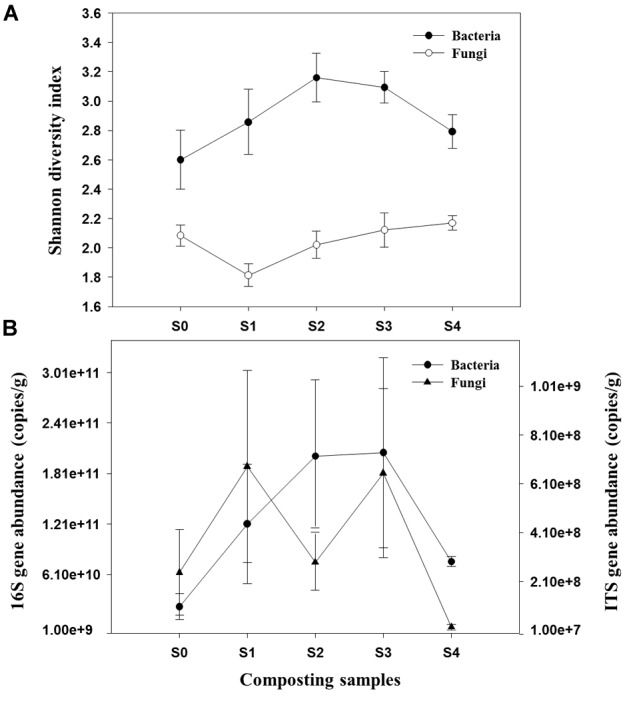
Shannon diversity index of bacterial and fungal communities **(A)** and abundance of 16S rRNA gene and ITS gene **(B)** during composting process.

A total of 534,988 high-quality bacterial and 1,905,601 fungal sequences were obtained after quality control filtering and potential chimeras were removed. Based on 97% sequence similarity, bacterial and fungal sequences were clustered into 272 and 321 OTUs, respectively. Rarefaction curves for observed bacterial and fungal OTUs reach the saturation platform, indicating that sequencing effort was sufficient to represent the entire bacterial and fungal populations (Supplementary Figure S3). One-way ANOVA analysis showed that microbial diversity and abundance changed significantly as the composting progressed (Supplementary Table S1). For bacteria, Shannon diversity index increased across both mesophilic and thermophilic stages and decreased during the maturation stage. However, peak value was observed during the 18th day of the composting process (Figure 1A). The fungal Shannon diversity index decreased at the early stage and was restored to the relative high level during the late stage of composting process. The qPCR assay that targeted the bacterial 16S rRNA gene and fungal ITS gene was used to determine the differences in bacterial and fungal abundance among different composting stages (Figure 1B). The change of bacterial 16S rRNA gene abundance had a similar trend with bacterial Shannon diversity index. In addition, the ITS gene abundance showed a significant decrease when temperature reached the thermophilic range, thus had an increasing trend at cooling stage.

### Bacterial and Fungal Communities

**FIGURE 2:**
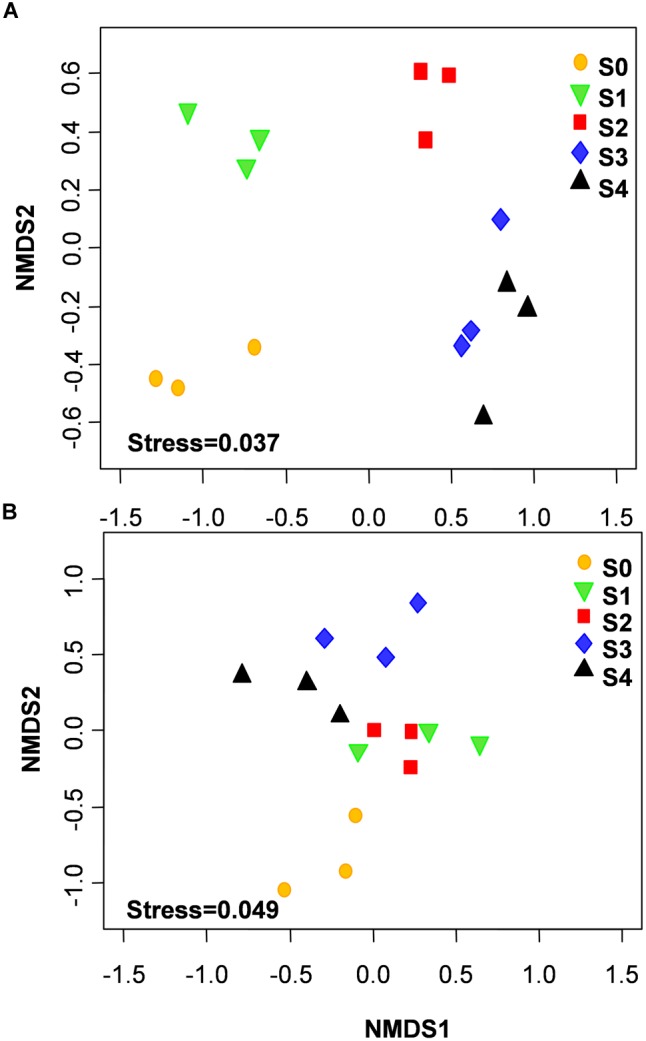
Non-metric multidimensional scaling (NMDS) of bacterial **(A)** and fungal **(B)** community structures was calculated with Bray–Curtis distances during the composting process.

**FIGURE 3:**
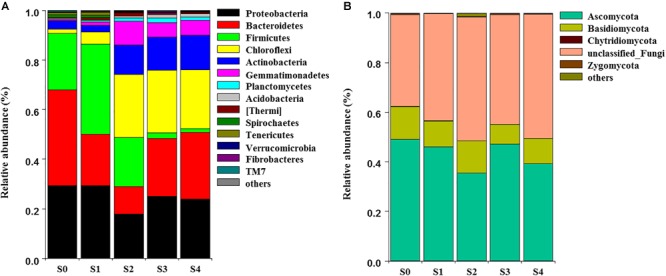
Dynamic changes of community composition of bacterial **(A)** and fungal **(B)** at the phylum level. The relative abundance of phylum was above 0.1% in at least one sample. Phyla with relative abundance less than 0.1% were defined as others.

**FIGURE 4:**
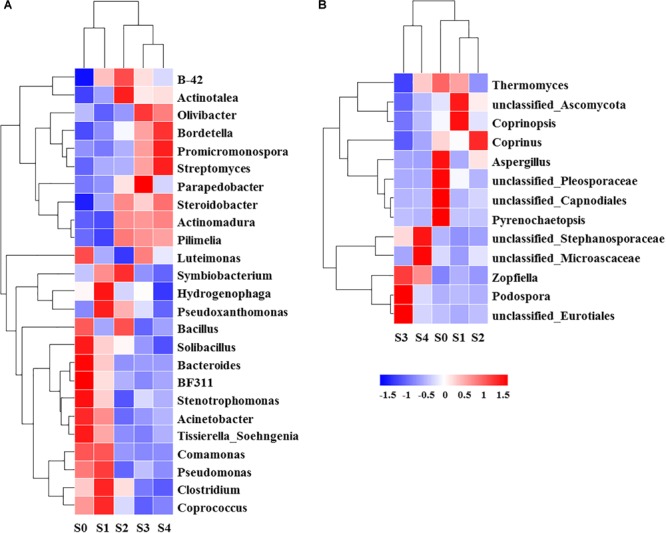
Relative abundance [log_2_ (x+1)] of top genera (relative abundance percentage >1%) of bacterial **(A)** and fungal **(B)** at different composting stages.

The NMDS showed that bacterial community structure was significantly different between the composting stages. However, the distance matrices of fungal community composition were closer than that of bacterial (Figure 2). The major bacterial phyla, which are Proteobacteria, Bacteroidetes, Firmicutes, Chloroflexi, Actinobacteria, Gemmatimonadetes, and Planctomycetes were observed in all samples. As shown in Figure 3A, Proteobacteria was the most abundant phylum, accounting for 25.1% of all OTUs. Within Proteobacteria, the β-proteobacteria was more abundant than α-proteobacteria, γ-proteobacteria, and δ-proteobacteria. Bacteroidetes (24.1%) was the second most frequent group, of which Bacteroidia and Flavobacteriia were the dominant classes. The other biggest group was Firmicutes (16.5%) with Clostridia as the most abundant class followed by Bacilli. At the initial stage, Bacteroidetes (38.6%) and Proteobacteria (29.4%) were the dominant phyla, followed by Firmicutes (22.7%), Actinobacteria (3.4%), Chloroflexi (1.6%). However, at the mesophilic phase, Bacteroidetes decreased by 17.9%, Firmicutes and Chloroflexi increased by 13.6 and 3.2%, respectively. Therefore, Firmicutes became the most abundant phylum in the mesophilic phase. With the rise in temperature, the abundance of Bacteroidetes, Proteobacteria and Firmicutes decreased by 9.7, 11.4, and 16.7% while Chloroflexi, Actinobacteria, and Gemmatimonadetes increased by 20.6, 9.3, and 8.6%. When temperature reached the thermophilic phase, Chloroflexi and Firmicutes became the major phyla followed by Proteobacteria and Actinobacteria. Compared with the thermophilic stage, the proportion of Bacteroidetes, Proteobacteria, and Actinobacteria increased during the cooling and maturation stages with Firmicutes and Chloroflexi decreased. At the genus level, the dominant genera (relative abundance >1%, Figure 4A) in composting were represented by *Acinetobacter* (9.4%), *Clostridium* (8.5%), *Bacteroides* (4.8%), *Luteimonas* (4.3%), *Comamonas* (4.0%), *Bordetella* (3.6%), and *Coprococcus* (3.5%). However, the relative abundance of these genera significantly decreased during the thermophilic phase. At the initial and mesophilic stages, *Acinetobacter* and *Bacteroides, Clostridium*, and *Coprococcus* were the dominant genera. The abundance of *Steroidobacter* and *Actinomadura* were dominant in the thermophilic phase, whereas *Olivibacter* and *Luteimonas, Bordetella*, and *Promicromonospora* were the dominant genera in the cooling and maturation phase.

Ascomycota (43.4%) was the most abundant fungal phylum among the different composting stages followed by Basidiomycota (10.9%) and Zygomycota (0.3%) (Figure 3B). Ascomycota was represented by Dothideomycetes and Sordariomycetes classes, as well as Agaricomycetes and Tremellomycetes were the dominant classes within Basidiomycota. The maximum abundance of Ascomycota and Basidiomycota was found during the initial stage while Ascomycota dominated the whole composting process. Ascomycota decreased at the mesophilic and thermophilic phases continuously, and then increased by 11.9% when temperature declined to cooling stage. The proportion of Basidiomycota decreased by 2.6% during the mesophilic phase, and later increased by 2.5% at the thermophilic phase. Although Zygomycota was observed in all samples, but there was no significant change in its abundance during the composting process. Unclassified_Ascomycota (34.7%), *Coprinus* (11.9%), *Zopfiella* (11.8%), and *Podospora* (6.4%) were observed to be the dominant fungal genera in this study (Figure 4B). The abundance of Unclassified_Ascomycota, *Zopfiella*, and *Podospora* decreased when temperature reached the thermophilic stage, and then increased during the late stage of composting. Unclassified_Ascomycota and *Coprinus* were dominated at the initial, mesophilic, and thermophilic stages. During the cooling and maturation phases, *Zopfiella* and Unclassified_Ascomycota were found to be the dominant genera, respectively.

**FIGURE 5:**
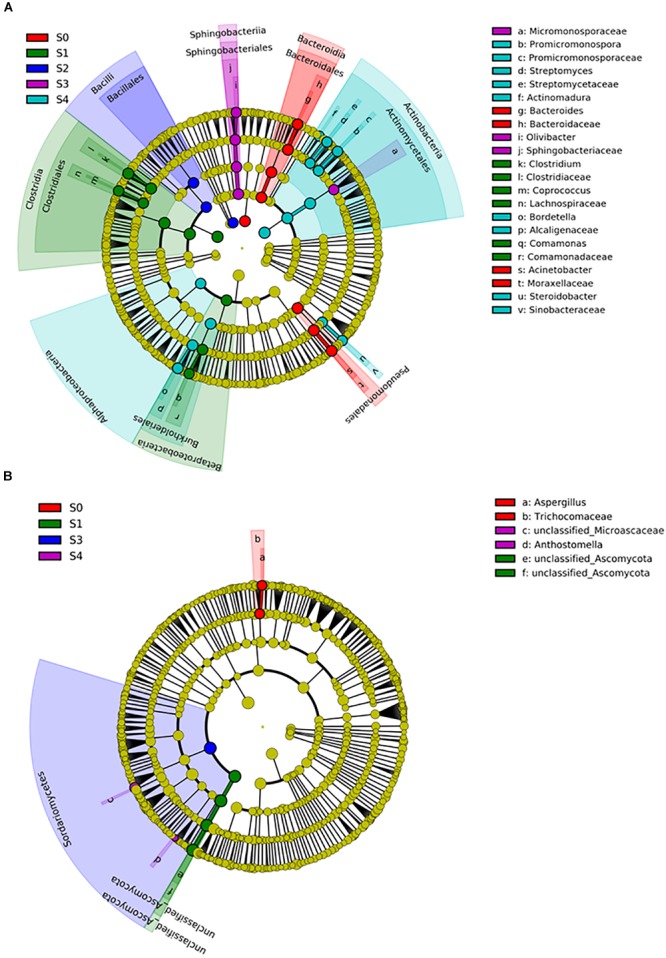
The differential phylogenetic distribution of bacterial **(A)** and fungal **(B)** in different composting stages. The linear discriminant analysis scores of ≥4. Circles indicate phylogenetic levels from phylum to genus, each circle’s diameter is proportional to the taxon’s abundance. The nodes of different colors represent the microbes that play an important role in the grouping represented by the color, yellow represent non-significant.

To determine the professional community/communities in samples, LEfSe was conducted to identify the groups that display significant differences among composting stages and these identified indicator groups were shown in cladogram. The bacterial and fungal taxa varied during the whole process. In the initial phase, LEfSe revealed that the indicator groups for bacteria were assigned to Bacteroidales and Pseudomonadales (Figure 5A). Clostridiales and *Comamonas* which belongs to Burkholderiales were the indicator groups in the mesophilic stage. Nevertheless, *Bordetella* belongs to Burkholderiales and Actinomycetales in which *Promicromonospora, Streptomyces*, and *Actinomadura* belongs, were also notable at the maturation phase. Bacillales and Sphingomonadales were the indicator groups for thermophilic and cooling phases, respectively. In addition, Trichocomaceae and *Aspergillus* (belongs to Trichocomaceae) were the fungal indicator groups during the initial phase (Figure 5B). In the mesophilic stage, Unclassified_Ascomycota was the indicator group. The Sordariomycetes, Anthostomella and Unclassified_Microascaceae were the indicator groups at cooling and maturation phases, respectively.

### Correlation Between Microbial Communities and Physicochemical Parameters

**FIGURE 6:**
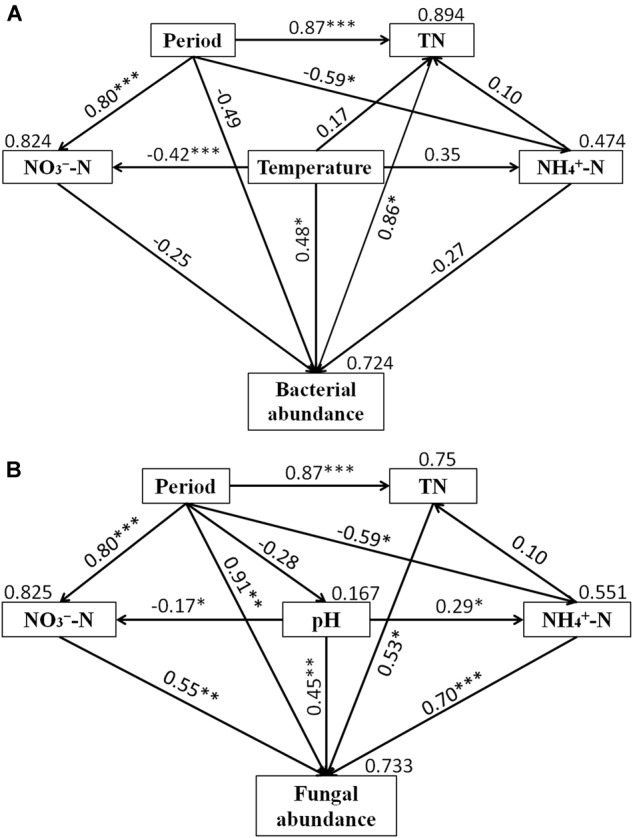
Structural equation models (SEMs) showing the effects of period, NH_4_^+^-N, NO_3_^-^-N, TN, temperature and pH on bacterial **(A)** and fungal **(B)** abundance. Values on the arrows represent the path coefficients. Values on the text box represent the proportion of variance explained for each variable. Lines with ^∗^indicate significant pathways (^∗∗∗^*P* < 0.001; ^∗∗^*P* < 0.01; ^∗^*P* < 0.05).

Structural equation model was performed to assess direct and indirect effects of environmental variables on microbial abundance (Figure 6). The SEM model for bacteria (χ^2^ = 1.992, *df* = 3, *P* = 0.574, RMSEA = 0.00, GFI = 0.956, AIC = 37.992) was able to explain 72.4% of the variation in bacterial abundance. For fungi, the SEM model (χ^2^ = 1.658, *df* = 3, *P* = 0.646, RMSEA = 0.00, GFI = 0.963, AIC = 37.658) explained 73.3% of fungal abundance. Temperature and TN had significant direct effects on the bacterial abundance, while period, NH_4_^+^-N and NO_3_^-^-N had no significant effect on the bacterial abundance. Furthermore, the indirect influence of period was observed on bacterial abundance. NH_4_^+^-N, NO_3_^-^-N, pH, TN and period had direct significant effects on fungal abundance. The indirect influence of period on fungal abundance was associated with the changes of NH_4_^+^-N, NO_3_^-^-N, and TN.

**FIGURE 7:**
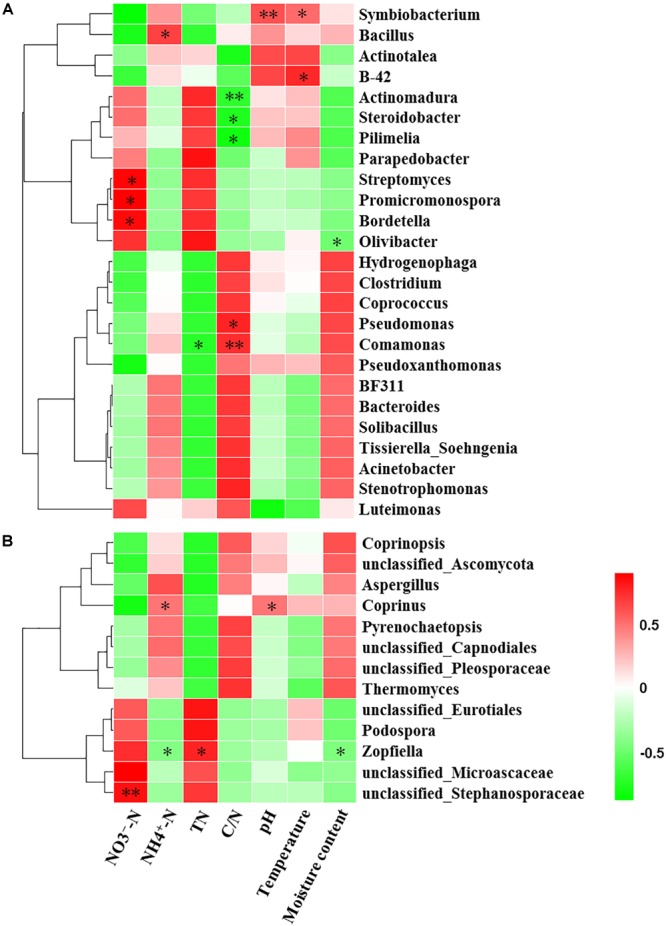
Pearson correlation heatmap of composting physicochemical parameters and the main genera (relative abundance >1%) of bacterial **(A)** and fungal **(B)**. TN, total nitrogen; C/N, the ratio of TC to TN; ^∗^Significant at *P* < 0.05; ^∗∗^Significant at *P* < 0.01.

Mantel test indicated that NO_3_^-^-N, NH_4_^+^-N, TN, C/N, temperature and moisture content significantly influenced bacterial community composition (Table 2). Moreover, partial Mantel test showed that NH_4_^+^-N, C/N, temperature and moisture content had significant relationship between bacterial communities. In addition, both the Mantel test and partial Mantel test indicated that TN and moisture content had a significant effect on fungal community structure. As shown in Figure 7A, Pearson correlation between the main genera and physicochemical parameters indicated that *Streptomyces, Promicromonospora*, and *Bordetella* were positively correlated and significant with NO_3_^-^-N, *Bacillus* was significant and positively correlated with NH_4_^+^-N. *Symbiobacterium* was positively correlated and significant with pH and temperature. *Pseudomonas* and *Comamonas* were significant and positively correlated with C/N, whereas, *Actinomadura, Steroidobacter*, and *Pilimelia* were negatively correlated with C/N. Unclassified Stephanosporaceae and Coprinopsis were significant and positively correlated with NO_3_^-^-N, NH_4_^+^-N, and pH (Figure 7B). *Zopfiella* was significant and positively correlated with TN while negatively correlated with NH_4_^+^-N and moisture content.

**Table 2:** Relationships between bacterial and fungal community compositions and environmental variables in the cow manure compost as revealed by Mantel test and partial Mantel test.

	Bacteria				Fungi			
Factors	Mantel test		Partial Mantel test		Mantel test		Partial Mantel test	
	*r*	*P*	*r*	*P*	*r*	*P*	*r*	*P*
NO_3_^-^-N	0.518	**0.003**	0.012	0.447	0.267	0.057	–	–
NH_4_^+^-N	0.184	**0.045**	0.195	**0.046**	0.195	0.137	–	–
TN	0.653	**0.001**	–0.315	1.000	0.275	**0.005**	0.418	**0.019**
C/N	0.743	**0.001**	0.588	**0.001**	0.003	0.441	–	–
pH	0.141	0.085	–	–	–0.168	0.890	–	–
T	0.188	**0.044**	0.342	**0.007**	–0.230	0.955	–	–
M	0.654	**0.001**	0.233	**0.023**	0.257	**0.001**	0.301	**0.041**

*Values in bold indicate significant correlations (*P* < 0.05). TN, total nitrogen; C/N, the ratio of TC to TN; T, temperature; M, Moisture content.*

## Discussion

The composition of the composting community proceeds in stages. During different stages, temperature, and nutrient availability vary and thus, affecting succession and abundance of microbe development. The present study used high-throughput sequencing to unravel the succession of bacterial and fungal communities and the relationships with the physicochemical factors driving these changes during cow manure composting. Our results showed that, the bacterial Shannon diversity index and abundance increased during the mesophilic phase and reached the peak value at the most productive stage of composting (thermophilic phase). This increase might be because of the availability of easily useable organic substances which produces the fastest growing microorganism, the bacteria. Hence, mesophilic bacteria, dominate initial decomposition by releasing heat from the breakdown of large amount of easily degraded organic matter. Similarly, several authors have reported a steady increase in both richness and diversity in diverse compost types as composting progressed through different phases (De Gannes et al., 2013; Wang X. et al., 2013; Ren et al., 2016; Wang et al., 2018a). Fungi Shannon diversity index and abundance noticeably reduced when temperature reached the thermophilic phase. This phenomenon agreed with past findings where authors reported reduced amount of fungi during the thermophilic stage of composting and resultant increase during the cooling stage (Langarica-Fuentes et al., 2014; Galitskaya et al., 2017). Our justification for this is that most compost inhabited fungi are not heat-tolerant, in contrast to bacteria. These observed results showed that bacterial and fungal communities respond differently to the temperature fluctuations, similar results were also observed during the composting of animal wastes (cattle and horse manure) (Tiquia, 2005; Huhe et al., 2017).

With the changes in physicochemical parameters and degradation of compost materials, composting process significantly affected microbial population (Insam and de Bertoldi, 2007). The NMDS revealed that microbial community compositions were significantly different between composting stages. Interestingly, distance matrices of fungal community compositions were closer than bacteria, indicating that a high similarity of fungal community compositions was shared between different composting stages. Diversity index analysis provided another basis for this result, because bacteria Shannon diversity index was higher than that of fungi in this study. In addition, bacteria community compositions in cooling and maturation phases were similar, suggesting that bacterial species are role specific and environment sensitive. This coupled with the nature and nutritional status in the composting system during the cooling and maturation phases could have resulted in that observation. This result agrees with previous study of Ren et al. (2016) who reported similarity of bacteria diversity at both the cooling and maturation stages during cow manure and rice straw composting.

Different microbial communities predominates the various composting phases, each of which being adapted to a particular environment (Ryckeboer et al., 2003). Qualified reads were assigned to different known taxa levels in order to identify the diversity of the bacterial and fungal communities during composting. Phyla Proteobacteria, Bacteroidetes, Firmicutes, Chloroflexi, and Actinobacteria were the most abundant bacteria in present study, this was in agreement with previous studies that they represented more than 90% of the total identified sequences (Partanen et al., 2010; Neher et al., 2013; Tortosa et al., 2016; Yin et al., 2017; Zhou et al., 2018). It indicated that these Phyla were the major players in degradation of organic materials during composting, this result was similar to other reports (Jurado et al., 2014; Awasthi et al., 2017). With temperature rising at mesophilic phase, the proportion of Bacteroidetes decreased, this agreed with the reports of Ren et al. (2016) and Ryckeboer et al. (2003). However, Firmicutes and Chloroflexi increased during the mesophilic phase, owing to the sugars, also easily degradative organic matters could supply enough nutrients through the decomposition and fermentation process (Liu et al., 2017) and Firmicutes has the ability of using carbohydrates effectively (Ariesyady et al., 2007). Chloroflexi and Firmicutes became the major phyla at the thermophilic phase, this may be explained by the fact that Firmicutes are endospore forming bacteria (de Hoon et al., 2010) and it has the tolerance to unfavorable conditions (Hartmann et al., 2014) or the ability of living under environmental stresses (Barnard et al., 2013). Other report even indicated that Firmicutes can grow at high temperature and widely distributed in the thermophilic phase of agricultural residues compost (Zhang et al., 2016). Compared with thermophilic phase, the proportion of Proteobacteria, Bacteroidetes, and Chloroflexi changed non-significant during the cooling and maturation stages and became the three major plyla. However, Firmicutes decreased significantly, this result was in accordance with other studies (Yamamoto et al., 2009; Ren et al., 2016). Actinobacteria increased gradually from thermophilic phase to the end of composting. Because Actinobacteria can degrade cellulose, hemicellulose and lignin are very effective in composting process (Steger et al., 2007). This result was similar to previous studies, which revealed that Actinobacteria was dominant in the later stages of composting (Insam and de Bertoldi, 2007; Steger et al., 2007). In this work, fungal communities had an obvious succession during the composting process, and this was in accordance with previous studies (De Gannes et al., 2013; Neher et al., 2013; Langarica-Fuentes et al., 2014). Ascomycota and Basidiomycota as the dominant fungal phylum, have been reported in composting of cow manure, food and garden waste and sewage sludge (Langarica-Fuentes et al., 2014; Wang et al., 2018a,b), which were also found in this study. Ascomycota decreased in the mesophilic and thermophilic stages, this might be owing to the fact that most fungi are mesophiles. Therefore, with the decreased temperature at the cooling stage, the abundant Ascomycota was observed. The reason for Ascomycota dominating all the phases of composting was that Ascomycota can secrete a variety of cellulose and hemicellulose degrading enzymes and efficiently utilize nutrients in compost (Singh et al., 2010). The abundance of Basidiomycota at thermophilic stage was higher than other stages, the result was similar to other research that a few Basidiomycota could grow well at high temperatures (Ryckeboer et al., 2003).

With the help of high sequence numbers, identified indicator OTUs were generally related to the microbe biomarkers (Wang et al., 2012). In this work, the bacteria had more indicators than fungi, of which Bacteroidales and Pseudomonadales were the major indicators at initial stage. This result was similar to previous study that Pseudomonadales were significantly more abundant in the raw materials than in other composting stages (Huhe et al., 2017). Bacillales was the indicator group for thermophilic phase, which was involved in the turnover of organic matter, including the extensive degradation of cellulose and lignocellulose residues (Pathma and Sakthivel, 2013; Wang Y. et al., 2013). Actinomycetales were notable in the maturation phase, which was known as saprophytes with the capability to degrade relatively recalcitrant plant polymers, such as lignin and suberin (Komeil et al., 2013; de Gonzalo et al., 2016). For fungi, *Aspergillus* was the indicator group during initial phase, which could engender degradation of organic matter (Awasthi et al., 2017). Other research reported that *Aspergillus* is a thermophilic fungal genus (Sebök et al., 2015), but no significant difference in the relative abundance of *Aspergillus* during thermophilic stage in this present study. The different growth environment might be the main reason for this result. Sordariomycetes, to which Anthostomella and Unclassified_Microascaceae belongs, was the indicator group at cooling and maturation phases, which has the ability to break down lignin and cellulose (Zhang et al., 2006). This result was in accordance with previous study that Sordariomycetes was enriched in the maturation stage (Huhe et al., 2017).

The change of physicochemical parameters has both direct and indirect influence on the activities of the microorganisms (Chandna et al., 2013). In this study, SEM analysis and Mantel test revealed that abundance and community composition of bacteria and fungi were significantly affected by different physicochemical parameters. A significant relationship was found between NO_3_^-^-N and NH_4_^+^-N with the fungal abundance but not with bacteria abundance, suggesting that growth of fungi was more sensitive to changes of NO_3_^-^-N and NH_4_^+^-N than that of bacteria. This might be as a result of NH_4_^+^-N being the preferred nitrogen source for most microorganisms (Geisseler et al., 2010) and the conversion of NH_4_^+^-N to NO_3_^-^-N (nitrification) is an important energy resource (John and John, 2018). However, NO_3_^-^-N and NH_4_^+^-N significantly influenced the bacterial community composition but not fungal community, this indicates that succession of bacterial community was more sensitive to the variation of NO_3_^-^-N and NH_4_^+^-N than that of fungal community. Pearson correlation showed that *Streptomyces, Promicromonospora, Bordetella*, and *Bacillus* were significant and positively correlated with NO_3_^-^-N and NH_4_^+^-N, while only Unclassified_Stephanosporaceae and Coprinopsis were positively correlated with NO_3_^-^-N and NH_4_^+^-N, respectively. This result was similar to other findings in which NO_3_^-^-N and NH_4_^+^-N were likely to influence, or be influenced by bacterial species but not fungal species during agricultural waste composting (Zhang et al., 2011). In addition, other study reported that NO_3_^-^-N and NH_4_^+^-N were the environmental factors which can affect the regulation of humic substance formation by the bacteria (Wu et al., 2017). Therefore, NO_3_^-^-N and NH_4_^+^-N were the important factors influencing the composting process. C/N had a significant effect on bacterial community composition in this study. This could probably be linked to the variation of C/N that is related to the degradation of organic matter by bacteria and thus compost stabilization. This agrees with the result of Wang et al. (2015) who reported that C/N significantly influences the bacterial species compositions in organic solid waste composting. Similarly, the significant Pearson relationship between *Pseudomonas, Comamonas, Actinomadura, Steroidobacter*, and *Pilimelia* with C/N was also observed in present study. Interestingly, TN significantly influenced the abundance and community composition of bacteria and fungi. This indicated that both bacteria and fungi played key roles in the transformation of nitrogen in composting. Therefore, rates of nutrient transformation and compost maturation are processes mainly sponsored by activities of microorganisms (Brown et al., 2013). A significant relationship between temperature and bacterial community composition was observed in present study. In addition, *Symbiobacterium*, isolating from thermophilic compost previously (Ueda et al., 2001), has a significant Pearson correlation with temperature in this study. Temperature is an important indicator of the process of composting and affects the microbial activity and determines the rate of organic matter decomposition. However, no significant relationship found between NO_3_^-^-N, NH_4_^+^-N, C/N and temperature with fungal community composition in this study. Notwithstanding, it did not signify that these factors did not influenced the fungal community composition only expresses that these factors influence on the fungal community composition is not significant in this study.

## Conclusion

Bacterial and fungal diversity and abundance changed significantly with composting process. High-throughput 16S rRNA/ITS gene sequencing indicated that the dominant phyla during composting included Proteobacteria, Bacteroidetes, Firmicutes, Chloroflexi and Actinobacteria of bacteria, Ascomycota and Basidiomycota of fungi. Bacteroidales, Pseudomonadales, Bacillales, and Actinomycetales were indicator group of bacteria, *Aspergillus* and Sordariomycetes were indicator group of fungi. Physicochemical parameters also varied during composting, our results indicated that NO_3_^-^-N, NH_4_^+^-N, C/N and temperature had significant effect on bacterial community succession only, but TN and moisture content affected both bacterial and fungal community significantly. TN, NH_4_^+^-N, NO_3_^-^-N, and pH had a significant effect on fungal abundance, while TN and temperature significantly affected bacterial abundance. Our findings extend understanding of the succession of microbial communities in cow manure and corn straw composting under natural conditions.

## Author Contributions

QM, WY, and XX designed and wrote the research. BX, LD, and XJ collected the samples. MM and SS did the DNA extraction and PCR amplification. XW, YH, and HZ did the composting. AB checked the text error of this manuscript.

## Conflict of Interest Statement

The authors declare that the research was conducted in the absence of any commercial or financial relationships that could be construed as a potential conflict of interest.
