# Supplementary material for: Microbial Community Succession and Response to Environmental Variables During Cow Manure and Corn Straw Composting
Source: Front Microbiol. 2019 Mar 18;10:529. doi: 10.3389/fmicb.2019.00529 (PMC6431636; doi:10.3389/fmicb.2019.00529)
Supplement: Supplementary file 1 [file Data_Sheet_1.docx]

**Succession of microbial communities and metabolic functions in cow manure and corn straw composting**

**Qingxin Meng^a^, Wei Yang^a^, Mengqi Men^a^, Ayodeji Bello^a^, Xiuhong Xu^a*^,Benshu Xu^a^, Liting Deng^a^, Xin Jiang^a^, Siyuan Sheng^a^, Xiaotong Wu^a^, Yue Han^a^, Haifeng Zhu^a^**

^a^ College of Resources and Environment, Northeast Agricultural University, Harbin 150030, China.

***Correspondence:**

Dr. Xiuhong Xu

E-mail: [xuxiuhong@neau.edu.cn](mailto:xuxiuhong@neau.edu.cn).

**Supplementary Figure 1** Changes of physicochemical characteristics during the cow manure composting. (A) pH; (B) Temperature; (C) Moisture content (M).

**Supplementary Figure 2** Changes of physicochemical characteristics during the cow manure composting. (A) NO_3_^-^-N; (B) NH_4_^+^-N; (C) total nitrogen (TN); (D) C/N.

**
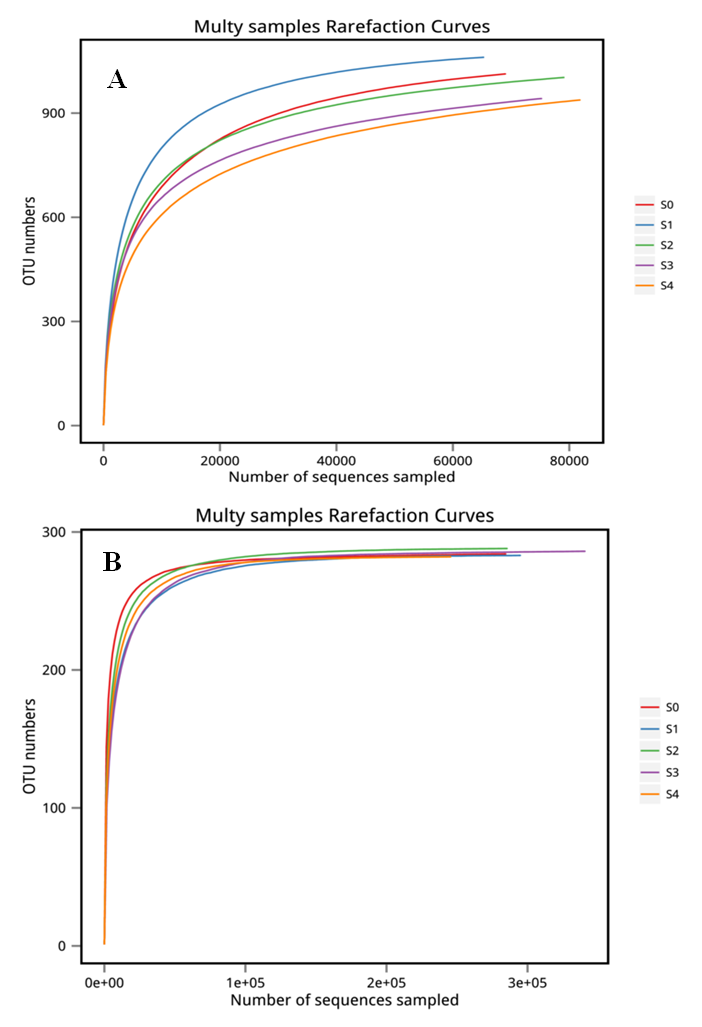
**

**Supplementary Figure 3** Rarefaction curves for the observed OTUs of bacteria (A) and fungi (B) among different composting stages.

**Supplementary Table 1** One-way ANOVA examining the effects of the cow manure compost phases on environmental variables, diversity indices and abundances of bacteria and fungi.

|  | **Phase** | |  |
| --- | --- | --- | --- |
| **Variables** | **F** | **P** | |
| NO_3_^–^–N | 64.885 | <0.001 | |
| NH_4_^+^–N | 8.309 | 0.003 | |
| TN | 20.428 | <0.001 | |
| C/N | 25.930 | <0.001 | |
| pH | 8.585 | 0.003 | |
| T | 383.416 | <0.001 | |
| M | 58.281 | <0.001 | |
| Bacterial Shannon index | 3.421 | 0.052 | |
| Fungal Shannon index | 5.130 | 0.016 | |
| 16S gene abundance | 3.593 | 0.046 | |
| ITS gene abundance | 3.745 | 0.041 | |

TN, total nitrogen; C/N, the ratio of TC to TN; T, temperature; M, Moisture content.

**PCR amplification thermal cycling condition**

The thermal cycling of MiSeq sequencing was as follows: (1) for bacteria, an initial denaturation at 94^◦^C for 4 min, 30 cycles of denaturation at 94°C for 40s, annealing at 56°C for 1 min, and extension at 72°C for 1 min, followed by a final extension at 72°C for 10 min; (2) for fungi, 94°C for 5 min, followed by 34 cycles of 94°C for 30 s, 54°C for 30 s and 68°C for 45 s, followed by a final elongation step at 72°C for 10 min. The quantification PCR cycling condition was 95°C for 5 min, followed by 40 cycles of 95°C for 5 s, 58°C for 30 s for 16S rRNA gene; 55°C for 30 s for ITS gene, and 72°C for 40 s.
